# The Monster in the Closet: Mothballs’ Link to Non-Hodgkin Lymphoma

**Published:** 2004-09

**Authors:** David J. Tenenbaum

Each year, according to the American Cancer Society, about 54,300 Americans are diagnosed with non-Hodgkin lymphoma (NHL), a cancer that originates in the lymph tissue, and about 19,400 people die from it. Several lines of evidence point to a possible association with pesticides. The incidence of NHL has roughly doubled since the 1970s, a few decades after a marked rise in U.S. household and agricultural pesticide use, and previous studies have found increases in chromosome aberrations and micronuclei in lymphocytes among pesticides applicators and some groups of farmers. This month, Ikuko Kato of Wayne State University and colleagues report an increased risk of NHL among New York State women with several types of pesticide exposure at home and on the job **[*EHP* 112:1275–1281]**.

In the retrospective case–control study, 376 women recruited at NHL diagnosis in the late 1990s were compared to 463 age-matched controls. Cases were identified through the New York State Cancer Registry; controls were found through the Health Care Financing Administration or state Department of Motor Vehicle records. All participants answered a survey regarding past exposure to pesticides of all types. Whereas most previous studies of the association between cancer and pesticide exposure have focused on occupational exposure, Kato and colleagues also asked about home exposure to products such as mothballs, flea and ant killers, head lice treatments, and house plant products.

The highest risk of NHL was associated with pesticide exposure that began between 1950 and 1969. The authors speculate that this relationship could reflect a long latency period for NHL, or the historic use of compounds that are particularly toxic and now banned, such as the organochlorine pesticides.

Among women who used pesticides at home, the 25% with the highest use had a 62% greater chance of developing NHL than women who never used such products. Also, NHL risk was 2.12 times greater among women who had worked at least 10 years on a farm where pesticides were used, compared with women who never worked on a farm.

When analyzing use of specific products, the researchers found a significant correlation between use of mothballs and NHL, although not a clear dose–response relationship. The authors note that the active ingredients of mothballs may be inhaled or absorbed through the skin during contact with treated clothing. Naphthalene and paradichlorobenzene, common active ingredients in mothballs, are among the most common toxic chemicals detected in indoor air. Earlier studies correlated these compounds with blood diseases including aplastic anemia and hemolytic anemia. *In vivo* and *in vitro* studies have shown cytotoxicity, genotoxicity, and carcinogenicity for naphthalene, paradichlorobenzene, and their metabolites.

The findings were limited by the possibility of recall bias—cancer patients may be more motivated than controls to remember pesticide exposure. However, a counterbalancing bias may have existed: exposed controls, upon learning that pesticides were one of the major research interests, may have been more interested in participating than nonexposed controls. Additionally, fewer than 50% of the subjects could recall the names of pesticides that had been used at their workplaces. While establishing a correlation between exposure to pesticides and disease does not prove that the pesticides caused disease, it does add detail to the growing picture of pesticide-caused hematologic toxicity, and suggests a need for further study of mothballs in particular.

## Figures and Tables

**Figure:**
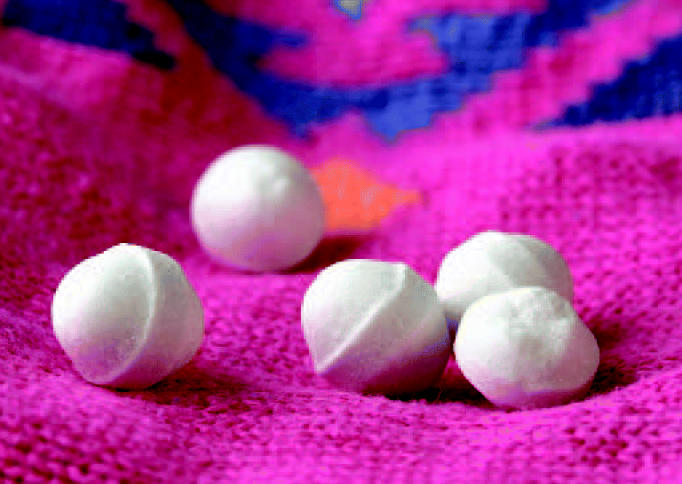
**Saves clothes, not health.** The naphthalene and paradichlorobenzene in mothballs may put those who use them at risk for non-Hodgkin lymphoma.

